# Effects of land use, habitat characteristics, and small mammal community composition on *Leptospira* prevalence in northeast Madagascar

**DOI:** 10.1371/journal.pntd.0008946

**Published:** 2020-12-31

**Authors:** James P. Herrera, Natalie R. Wickenkamp, Magali Turpin, Fiona Baudino, Pablo Tortosa, Steven M. Goodman, Voahangy Soarimalala, Tamby Nasaina Ranaivoson, Charles L. Nunn

**Affiliations:** 1 Duke Lemur Center SAVA Conservation, Durham North Carolina, United States of America; 2 Evolutionary Anthropology, Duke University, Durham North Carolina, United States of America; 3 Duke Global Health Institute, Duke University, Durham North Carolina, United States of America; 4 Unité Mixte de Recherche Processus Infectieux en Milieu Insulaire Tropical-UMR PIMIT (Université de la Réunion, CNRS 9192, INSERM 1187, IRD 249), Sainte-Clotilde, Réunion Island, France; 5 Association Vahatra, Madagascar; 6 Field Museum of Natural History, Chicago, Illinois, United States of America; 7 Mention Zoologie et Biodiversité Animale, Domaine des Sciences et Technologie, Université d’Antananarivo, Madagascar; University of Connecticut Health Center, UNITED STATES

## Abstract

Human activities can increase or decrease risks of acquiring a zoonotic disease, notably by affecting the composition and abundance of hosts. This study investigated the links between land use and infectious disease risk in northeast Madagascar, where human subsistence activities and population growth are encroaching on native habitats and the associated biota. We collected new data on pathogenic *Leptospira*, which are bacteria maintained in small mammal reservoirs. Transmission can occur through close contact, but most frequently through indirect contact with water contaminated by the urine of infected hosts. The probability of infection and prevalence was compared across a gradient of natural moist evergreen forest, nearby forest fragments, flooded rice and other types of agricultural fields, and in homes in a rural village. Using these data, we tested specific hypotheses for how land use alters ecological communities and influences disease transmission. The relative abundance and proportion of exotic species was highest in the anthropogenic habitats, while the relative abundance of native species was highest in the forested habitats. Prevalence of *Leptospira* was significantly higher in introduced compared to endemic species. Lastly, the probability of infection with *Leptospira* was highest in introduced small mammal species, and lower in forest fragments compared to other habitat types. Our results highlight how human land use affects the small mammal community composition and in turn disease dynamics. Introduced species likely transmit *Leptospira* to native species where they co-occur, and may displace the *Leptospira* species naturally occurring in Madagascar. The frequent spatial overlap of people and introduced species likely also has consequences for public health.

## Author summary

Many neglected tropical diseases have reservoirs in wildlife. The effects of human activities on wildlife include changes in species abundance, community composition, and the transmission dynamics of parasites. Introduced species, especially black rats (*Rattus rattus*) are known to transmit zoonotic diseases among wildlife species and people. *Leptospira*, a water-borne bacterium that infects wildlife and people, is an important pathogen in the tropics, and in Madagascar, multiple strains and hosts have been identified. We tested how land use gradients in a forest-frontier agricultural system affect the composition of small mammal communities, and in turn the prevalence of *Leptospira*. We investigated 11 species of small mammals, including native rodents and tenrecs, as well as introduced rodents and shrews, in old growth forest, forest fragments, agricultural fields, and in a village. *Leptospira* prevalence and infection risk were highest in introduced species compared to native species and lower in forest fragments. The results highlight how the introduction of non-native species affects the variation in disease prevalence in small mammals, with potential consequences for spill-over to people.

## Introduction

The global biodiversity crisis has far-reaching effects on human well-being, including changes in disease prevalence in wildlife and people [1,2]. Loss of biodiversity can increase parasite prevalence due to a phenomenon termed the “dilution effect” [3,4]. In the case of Lyme disease in the USA, for example, decreased mammal diversity due to habitat fragmentation led to higher incidence of the pathogen, *Borrelia*, in wildlife [3]. In this case, habitat fragmentation favored an increased abundance of the reservoir host, the white-footed mouse, *Peromyscus leucopus* (family Cricetidae), which in turn increased abundance and transmission of the tick vector [3]. This example and others [5] illustrate the important associations between community ecology and disease transmission dynamics.

A contrasting “amplification effect” is expected when between-species disease transmission increases with host diversity [4]. For example, infection with tick-borne *Anaplasma*, a pathogen that causes rickettsial disease, varied among small mammal species and vegetation types, but overall was higher in habitats where mammalian diversity was higher [6]. Thus, the nuances of the biological system affect the predicted trajectory of disease transmission in ecological communities.

Linking changes in biodiversity to infectious disease is fundamental to understanding the impact of global changes on the functioning of ecosystems [7]. Alterations in wildlife communities due to habitat degradation include reduced abundance of native species and the introduction of exotic species. Introduced species, which often become invasive, can alter the disease dynamics of biological systems and the risks for wild and domestic animals, as well as humans [7–11]. In disease-mediated invasion, introduced species bring pathogens to which they are immune, but to which native species are highly susceptible [12]. The decline of red squirrels (*Sciurus vulgaris*, family Sciuridae) in the UK after the introduction of American grey squirrels (*S*. *carolinensis*) and their parapox virus is one such example [13,14]. There are many species threatened with extinction due in large part to novel diseases from introduced species [15]. Entire ecosystems may be affected when introduced diseases spill over to keystone species [16].

Despite the important role introduced species play in altering local community composition and disease transmission dynamics, parameters that are key to understanding the mechanisms of these dynamics are still poorly understood [7]. For example, introduced species have different effects on overall community diversity across systems, sometimes causing reduced diversity due to competitive exclusion, but often increasing diversity as well [17,18]. In light of the dilution and amplification effects, these changes in community diversity due to introduced species will have cascading effects on disease dynamics. If the introduced and native species are competent hosts, then the addition of introduced species and increase in diversity leads to a larger pool of potential hosts and is predicted to result in an amplification effect. In contrast, if native species are noncompetent hosts, then higher diversity should lead to a dilution effect. Although some studies have experimentally manipulated animal populations and measured the effect of such manipulations on pathogen prevalence [19], it is crucial to address the impact of land use on disease transmission in real human-modified natural systems.

While research on biodiversity and disease relationships has largely focused on parasites transmitted through vectors or direct contact, less is known about the effects of biodiversity on parasites that are transmitted through the environment, i.e., in soil and water. One important pathogenic bacterium of global importance that is environmentally transmitted is *Leptospira*, which causes the diseases leptospirosis. Across the tropics, leptospirosis is among the most important neglected diseases [20], estimated to result in over 1 million human cases and almost 60,000 deaths per year [21]. Rats (genus *Rattus*, family Muridae) are known to be important reservoirs for *Leptospira* because they are asymptomatic and retain the bacteria in their renal tissue, shedding living microbes into the environment through urine [22]. *Leptospira* is predominantly water-borne, but viable *Leptospira* can persist in soil for months [23], and contact by people with contaminated soil and water sources can lead to infection. Infection is often characterized by mild flu-like symptoms but it can also lead to the life-threatening Weil’s disease, which is characterized by jaundice, pulmonary hemorrhagic fever and multi-organ failure [20]. To date, there have been few investigations of how variation in human land use affects the prevalence of *Leptospira* across host species. In southeast Asia, two *Leptospira* species differed in the infection and prevalence in rodents according to habitat, with one (*L*. *borgpetersenii*) found in forest, flooded and dry habitats while the other (*L*. *interrogans*) was restricted to forest and flooded environments [24]. Therefore, it is likely that habitat type affects the dynamics of *Leptospira* transmission.

Madagascar is renowned for its biodiversity, and all native terrestrial mammals are endemic, including rodents and tenrecs [25]. Native non-primate mammal communities are often diverse, with up to 20 sympatric species in a community and over 69 species known from the island [26,27]. For mammals, rodents are often a diverse component of communities, and are important reservoirs of zoonotic parasites globally [28]. There are now several known cases of pathogen spillover from introduced mammals to primates in Madagascar, such as canine heart worm (*Dirofilaria immitis*) in mouse lemurs (*Microcebus lehilahytsara*, family Cheirogaleidae), and enteric viruses and protozoa in several lemur species [10,29,30]. Endemic species can be negatively impacted by introduced small mammals via parasite spillover [25,31–33], which has been proposed to cause extinctions via introductions of new parasites in other areas of the world [34]. In addition to affecting native mammalian species, mammal-borne diseases, including leptospirosis, represent potential health risks for humans [35–37]. Although human leptospirosis is a major zoonotic disease with highest incidence in tropical countries, pathogenic *Leptospira* were not known from Madagascar until recently [35–38]. Given the environmental mode of transmission through soil and water, people and animals in rural Madagascar are likely to be highly exposed to *Leptospira* because a predominant form of agriculture is flooded-field paddy rice [39]. Little is known, however, about how *Leptospira* infection varies in relation to human land use. Native and introduced mammals in Madagascar have been reported to shed *Leptospira* in their urine, and domesticated animals and humans are exposed to infection [35,36,38,40]. Importantly, introduced small mammals are mostly infected by the globally-distributed *L*. *interrogans* and *L*. *borgpetersenii*, while endemic tenrecs (family Tenrecidae) host an endemic species, *L*. *mayottensis*, though infection with the widespread species *L*. *kirschneri* has also been documented [35,37,41]. Endemic Malagasy rodents had a prevalence of up to 25%, and were co-infected with *L*. *interrogans*, *L*. *mayottensis*, *L*. *borgpetersenii*, as well as previously undescribed lineages suggesting deep co-evolution between small mammals and leptospires [37,41,42]. Recently, sampling of livestock in Madagascar also revealed mixed infections and high prevalence of *Leptospira*, including *L*. *kirschneri*, *L*. *interrogans*, and *L*. *borgpetersenii* [40]. *Leptospira* haplotypes found in domestic animals were clearly related to those described in small mammals from Madagascar, as well as in livestock from Tanzania, Réunion, and Brazil [40].

Altogether, our current understanding of *Leptospira* in Madagascar shows that the complex community of small mammals and domestic animals is host to a diversity of pathogenic *Leptospira* composed of introduced (cosmopolitan) and endemic lineages and species [43]. Madagascar is therefore a model system to address how habitat degradation affects complex host-parasite interactions and in turn impacts public health.

We investigated the effects of anthropogenic land use in the native and introduced small mammal communities around a protected area in northeast Madagascar, where human subsistence activities and population growth are encroaching on the remaining moist evergreen forest in the area. Specifically, we collected data on *Leptospira* in wild mammals to determine the relationship between human land use, wildlife species diversity, and infection. Given the potential for small mammals to vary in their importance as reservoirs of infectious diseases [44,45], we hypothesized that human activities influence *Leptospira* prevalence via changes in small mammal diversity and abundance–and especially variation in the presence of introduced rodents (family Muridae) and shrews (family Soricidae). We tested the hypothesis that *Leptospira* prevalence was related to small mammal diversity; a positive association between prevalence and higher species diversity would be consistent with an amplification effect, while a negative association would be consistent with a dilution effect [4,9]. We also investigated whether prevalence was positively related to mammalian abundance, the proportion of individuals captured that were introduced species, and whether prevalence varied across habitat types, predicting highest prevalence in flooded rice fields (due to water-borne transmission) and lowest prevalence in forest habitats. Further, we tested whether introduced mammal species had a higher probability of *Leptospira* infection than native species, if infection varied among sexes, and if individuals captured in flooded rice fields had higher probability of infection than those caught in other habitat types. To determine if there were ecological predictors of *Leptospira* infection, we also tested whether the probability of infection varied among ecological guilds in terms of general diet and locomotor classifications. This is the first investigation that specifically tests the effects of introduced species, land use, and ecological niche as predictors of *Leptospira* infection in Madagascar.

## Methods

### Ethics statement

All procedures were approved by IACUC at Duke University (protocol number A002-17-01, 2017–2018), and by Malagasy authorities (No. 289/17 & 146/18—MEEF/SG/DGF/DSAP/SCB.Re, June-December 2017 and June-December 2018).

### Sampling sites and sampling design

**Fig 1:**
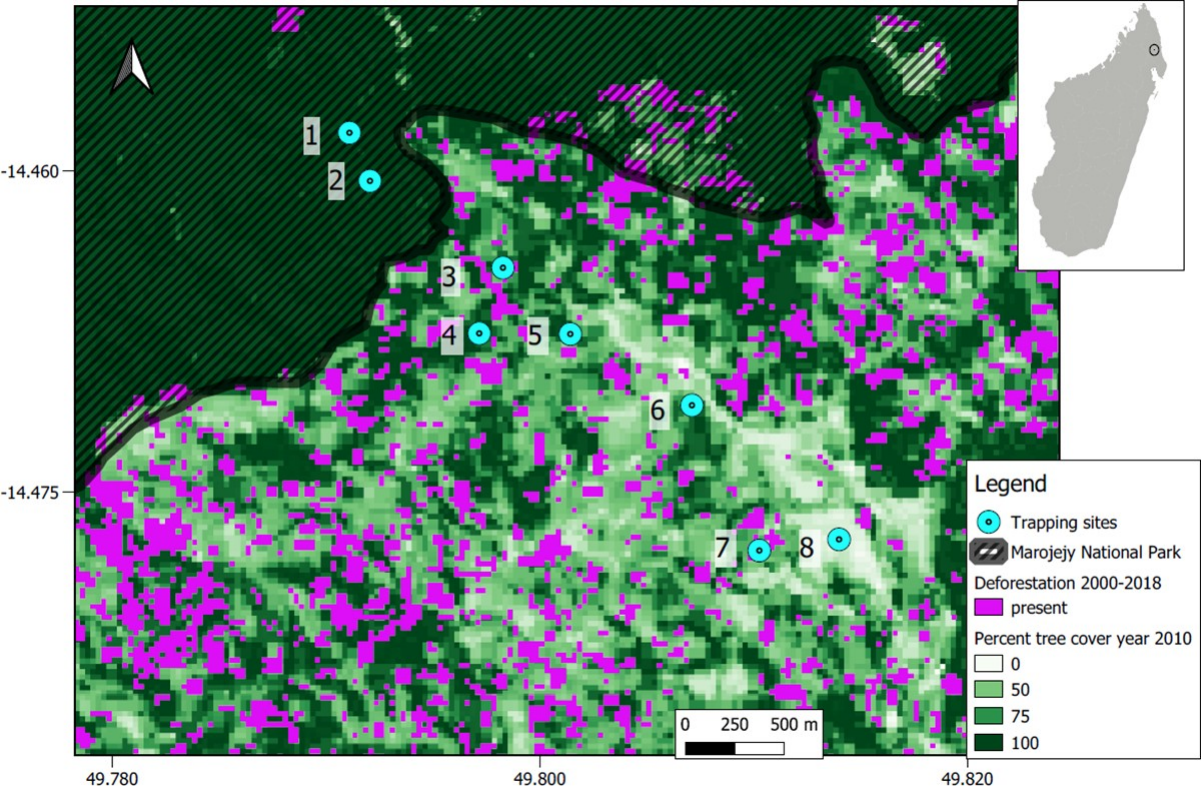
Map of study sites in northeast Madagascar. The locations of the eight trapping sites are overlaid on a map of the percent forest cover as of 2010, as well as deforestation between 2001 and 2018 (30m resolution, Source: Hansen/UMD/Google/USGS/NASA [46]). The border of the Marojejy National Park is shown. The habitats at trap sites are as follows: 1–2 = forest, 3–4 = fragments, 5–6 = flooded rice fields, 7 = swidden agriculture, 8 = Mandena village. The inset map indicates with a circle the general position of the study site in northeastern Madagascar.

This study was conducted in the northeast of Madagascar, inside and around Marojejy National Park (Fig 1). The park encompasses natural moist evergreen forests ranging from low elevation to high mountain peaks over 2000 m, while the land around the park has been cleared for agricultural production, including swidden agriculture, for rice, beans, and cassava, as well as fruit tree groves, vanilla plantations, flooded paddy rice, and settlements. Data were collected in and near the village of Mandena and the adjacent agricultural fields and forests.

We sampled small mammals from eight plots of land representing five habitat types: swidden hillside agriculture for rice and cassava (n = 1 plot), lowland flooded rice fields (n = 2, with varying amounts of mixed crops), secondary forest fragments (n = 2), the forest interior within the park (n = 2), and in homes in the village (n = 1 village, 41 households, Fig 1). Given that *Leptospira* is typically water-borne, variation in water sources at these sites likely played a role in transmission. The lowland flooded rice fields had the most standing water and the closest streams and canals, but the fields were not always flooded during sampling, and were more often sampled soon after being drained and rice was harvested. The forest sites had small streams within 100m of the trapping grids, while forest fragments were situated on hills and slopes above streams or a river within 100m. The swidden plot was a dry sloping hillside after rice had been harvested and there were also mixed plantations of cassava, with lowland rice fields below. Within the village, the closest water source is the Manantenina river, which is ~50-400m from the households.

These plots were sampled during three field sessions, twice in the transitional period between wet and dry seasons (June-August 2017 and 2018), and once in the transitional period between dry and wet seasons (November-December 2017). It was not possible to sample all plots during each season due to logistical constraints. We sampled each site for 5–15 consecutive days using two methods of nonlethal traps simultaneously (S1 and S2 Tables). Sampling effort varied among sites in relation to the abundance of species at those sites. Species in forest sites had low abundance, which required more sampling to acquire sufficient sample sizes and reliably estimate species diversity. First, we established a 90 m X 90 m grid of 100 live traps, including 75 Sherman (H. B. Sherman Traps, Inc., Tallahassee, Florida, model LFA and XLK), 10 Tomahawk (Tomahawk Live Trap, Hazelhurst, Wisconsin, model 201), and 15 Havahart (Havahart, Lancaster, Pennsylvania, model 1079-B) at 10 m intervals set on the ground and baited with peanut butter and banana. Second, we set two pitfall lines within 15–20 m ground distance of the grid, each line being 100 m long, with 15 liter buckets every 10 m (11 pitfall traps per line), dug in the ground to rim level, and a 0.80 m high plastic sheet stapled to wooden stakes to create a vertical barrier fence that bisected each bucket. The total cumulative capture effort during the different field sessions was 11,035 trap-days for traps and 2,235 trap-days for pitfalls.

Captured small mammals included introduced Muridae rodents and Soricidae shrews, as well as endemic rodents (subfamily Nesomyinae) and tenrecs (family Tenrecidae). Species could be broadly classified as primarily granivores or insectivores, and primarily terrestrial or both terrestrial and scansorial, based on published literature [25]. Preliminary species identifications of all captured animals were made in hand, including a series of measurements. Between five and 10 captured individuals of each endemic species were euthanized and collected for voucher and tissue specimens. A subset of individuals of endemic species were released at the site of capture to avoid overharvesting rare species (e.g., *Eliurus*, *Microgale*). All introduced species (murids and soricids) were euthanized. Euthanasia was performed using cervical dislocation for most animals, but for the larger tenrecs (*Setifer* and *Tenrec*), a lethal dose of xylazine was administered via intermuscular injection. For euthanized mammals, we collected kidneys, urine, and blood, with all material stored in 75% ethanol. All specimens were deposited in the Département de Biologie, Université d’Antananarivo (UADBA) collection after verification of species identification by VS and SMG.

### Tissue processing and molecular analyses

All tissue samples were immediately placed in 75% ethanol in the field, shipped to the Institut de Recherche pour le Développement lab facilities on La Réunion (France), and stored at -20°C. Kidney samples were rehydrated overnight and 20 mg of tissue was used for total nucleic acid (RNA/DNA) extraction using a Qiagen HT robot and Cador pathogen kits (Qiagen, Courtaboeuf, France). *Leptospira* detection was carried out through a probe-based real-time PCR [44]. The leptospire load was quantified based on the cycle threshold (Ct) value from the real-time PCR, with Ct values ≤ 40.00 considered a positive sample [47]. Sequencing of *secY* was attempted on all positive samples using standard primers [48,49] and with a set of primers optimized to amplify *Leptospira* prevailing in southwestern Indian ocean islands [37]. The *secY* locus is among the most polymorphic marker within the published multilocus sequence typing schemes and hence suitable for *Leptospira* species identification and epidemiological studies [50]. Amplicons were sequenced on both strands (Genoscreen, Lille, France) and allele identification was carried out through the *Leptospira* PubMLST database (https://pubmlst.org/leptospira/).

### Statistical analyses

We used a linear mixed-effects model to investigate the predictors of *Leptospira* prevalence in our sample. These predictors included small mammal species diversity (Shannon diversity index), habitat type (forest as reference level, compared to fragment, rice field, swidden, and village), abundance (capture rate per 1000 trapping attempts), diet and locomotor guild, and the proportion of individuals captured that were introduced species. We tested if these variables were significant predictors of *Leptospira* prevalence per species of small mammals collected in a plot. We used the “nlme” package [51] in the R statistical environment [52]. We included the study species as random effects to control for species-level variance. We also included the plot identity as a random effect because four plots were resampled during two seasons in an effort to capture seasonality, while for four plots, we were only able to sample once during the transition to the dry season. This analysis was restricted to those species for which there were multiple individuals captured across plots.

To investigate the factors that predicted the probability of infection with *Leptospira* at the individual-level, we conducted logistic regressions to predict infection status based on the habitat type, whether the individual was an introduced or native species, diet and locomotor guilds, abundance, the proportion of individuals captured in the plot that were introduced species, and the sex. We included latitude as a means to capture the gradient from the forest in the north of the study site through the agricultural lands to the village in the south (Fig 1). We used mixed model logistic regression, including the trapping site as a random variable in the “lme4” package [53] in R.

To account for the potential effect of phylogenetic relatedness among host species on prevalence and infection probability, we also used phylogenetically-informed analyses implemented in the package “MCMCglmm” [54], using one of the trees drawn at random from the distribution of near-complete mammal phylogenies [55]. This Bayesian implementation used weak (nu = 1) and diffuse (V = 0.02) priors on the variance components, after comparing model performance with varying prior values, and four chains were run for 5X10^6 million generations, discarding the first one thousand as burn-in, and sampling every 500 generations. Convergence and stationarity were assessed based on potential scale reduction factors close to 1, and effective sample sizes > 200.

We compared the effects of different predictor variables on *Leptospira* prevalence and infection using model-comparison and model averaging. Specifically, we used the “dredge” function in the R package MuMIn [56] to compare the full model with all possible combinations of variables and assessed their support based on the Akaike information criterion corrected for small sample sizes (AICc) for the likelihood-based approaches. The model coefficients were then averaged based on the Akaike weights to estimate the effects of each predictor.

## Results

**Fig 2:**
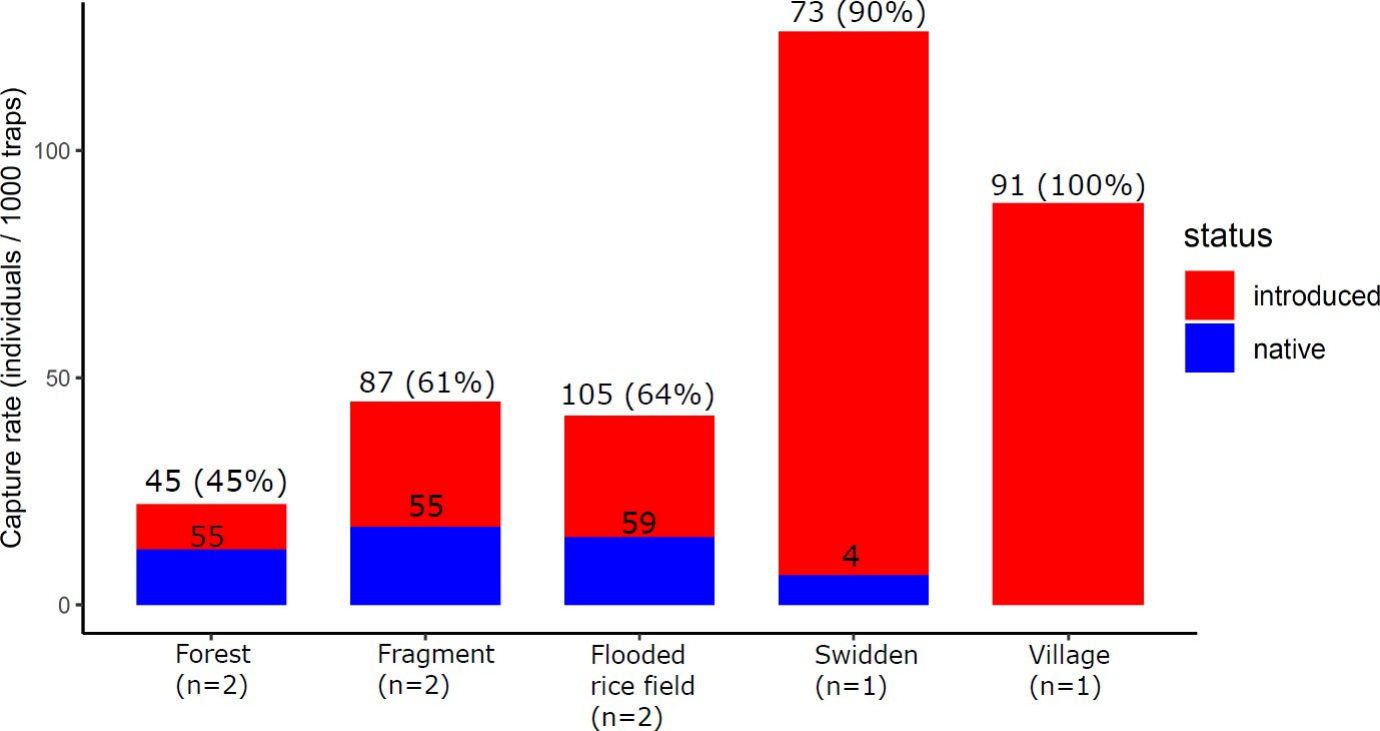
Variation in the relative abundance of introduced and native small mammals across habitat types (number of sampled sites indicated). The y-axis represents capture rate (number of individuals captured per 1000 trap-nights), while the numbers on bars represent the number of captured individuals.

We obtained biological samples from 530 small mammals. We captured endemic tenrecs (*Microgale brevicaudata*, *M*. *prolixacaudata* [formerly *longicaudata*], *Setifer setosus*, and *Tenrec ecaudatus*), endemic nesomyine rodents (*Eliurus ellermani* [formerly *tanala*], *E*. *webbi*, and *Nesomys rufus*), introduced murid rodents (*Rattus rattus* and *Mus musculus*), and introduced soricid shrews (*Suncus etruscus* and *S*. *murinus*). On the basis of external measurements and phenotypic characters, we assigned all of the *Rattus* specimens to *R*. *rattus*. Comparisons of their capture rates illustrated that relative abundance varied across sites (Fig 2, S1 Fig, S2 and S3 Tables). The relative abundance and proportion of introduced species was highest in the anthropogenic habitats, while the relative abundance of native species was highest in the forested habitats within the park and in fragments. While most nesomyine rodents and tenrecs were forest-dependent, there were some exceptions. Specifically, spiny tenrecs of the subfamily Tenrecinae (*Tenrec* and *Setifer*) and shrew tenrecs of the subfamily Oryzorictinae (*Microgale brevicaudata* and *M*. *prolixacaudata*) also occurred outside of natural forest.

**Fig 3:**
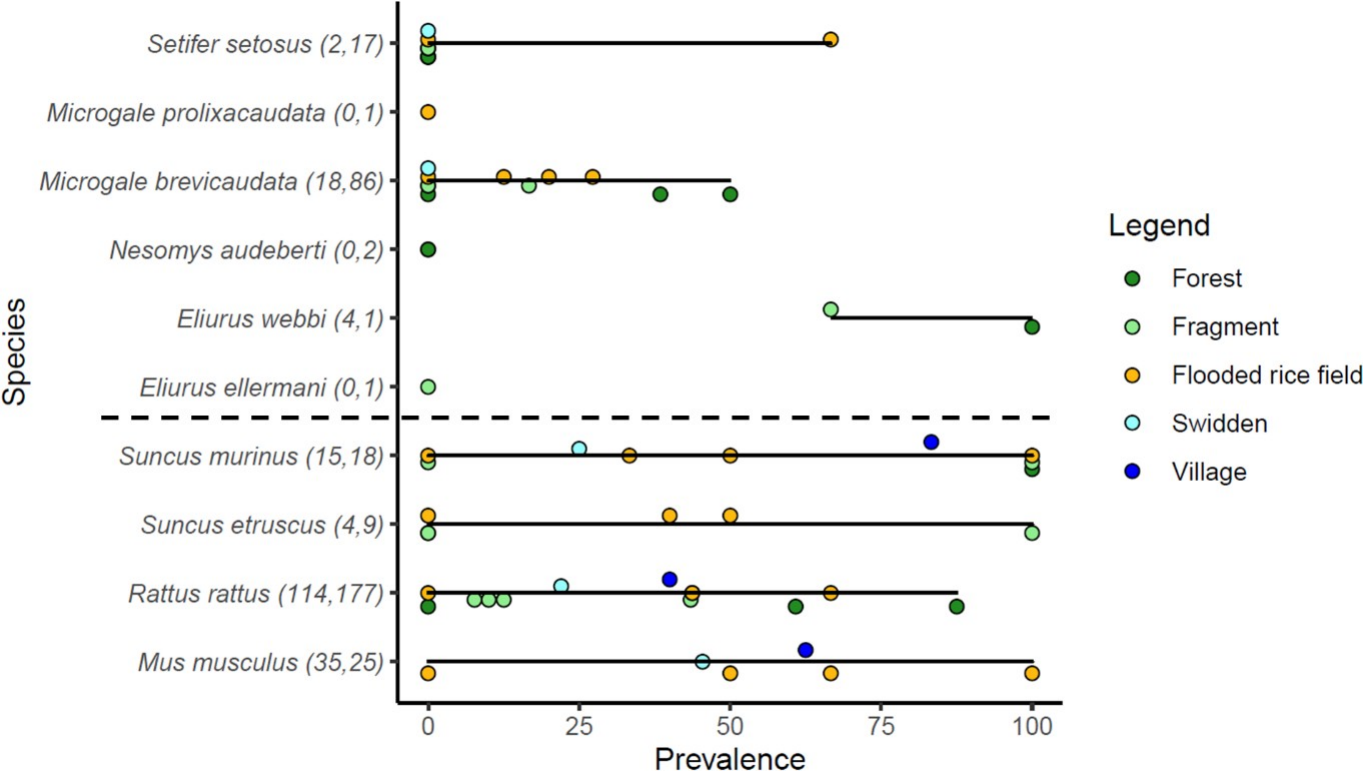
Prevalence of *Leptospira* infection in each species across habitat types. Each point represents the prevalence of *Leptospira* infection at a site and season, color coded by habitat type. The black lines represent the range in the values. The last four species below the dotted line are introduced to Madagascar. Points are offset slightly to allow visualization for points that overlap. The numbers in parentheses next to species labels represent the number of samples that tested positive and negative, respectively.

Altogether, of 530 animals, 192 tested positive for *Leptospira* based on qPCR results (Fig 3). Of the individuals that tested positive, 168 were introduced species (87.5%), while 24 were native (12.5%). Thirty-nine *secY* sequences could be obtained from the positive samples using standard and degenerated primers [37] (GenBank accession numbers MT81160—MT81194) Only two distinct *secY* haplotypes were found in these sequences, corresponding to *L*. *interrogans* and *L*. *kirschneri*. None of the positive samples were identified as *L*. *mayottensis*, which was previously isolated and/or identified in Malagasy small mammals [37,42]. Most sequences were obtained from introduced rodents and shrews, with the exception of one endemic spiny tenrec (*Setifer setosus*, see below). The *secY* sequences obtained from infected rats all corresponded to a single *L*. *interrogans secY* haplotype (23 sequences) that did not correspond to any reference allele on the PubMLST database. However, this allele was identical to *L*. *interrogans* sequences originating from Kuwait (KF770704.1) and Thailand (JF509194.1). Eleven out of 12 *Leptospira* sequences from *Mus musculus* (12 sequences), one *Suncus etruscus* and one *S*. *murinus* specimens were infected by *L*. *kirschneri* represented by the single secY^3^22 haplotype. The sequences obtained from the remaining *M*. *musculus* (N = 1) and *Setifer setosus* (N = 1) specimens corresponded to the same *L*. *interrogans secY* allele found in all genotyped rats.

Infected animals were captured in all sites and habitats, and *Leptospira* prevalence varied across habitats (S4 Table). Prevalence was significantly higher in the introduced species compared to the native species (Table 1). There were no statistical relationships between prevalence and small mammal diversity, measures of abundance, niche, or habitat type (Table 1). The full model explained 31% of the variance in the prevalence data. The results were similar using the phylogenetically-informed analyses (S5 Table).

**Table 1:** Effects of predictor variables on *Leptospira* prevalence in small mammals. Model-averaged results of linear mixed-effects models to explain variation in prevalence (natural log+1 transformed) based on the fixed factors (variables), controlling for random effects due to sites, which were repeated over time, and species. Significant effects are indicated with italics. These results are based on the models that have a cumulative AIC weight of ≥ 0.95. Note: the following levels were set as the reference (baseline) for comparison: Forest, Granivore, Terrestrial, Female, and Introduced. Coefficients are based on z-score transformation for continuous variables.

Variable	Coefficient (SE)
*Native*	*-1*.*52 (0*.43)*
Flooded rice field	0.11 (1.83)
Fragment	-0.46 (1.74)
Swidden	-1.05 (2.58)
Village	0.03 (2.91)
Proportion introduced	0.79 (2.69)
Shannon diversity	-1.34 (1.49)
Abundance	0.29 (0.23)
Terrestrial/Scansorial	0.09 (0.56)
Insectivore	-0.09 (0.53)

* *p = 0*.*001*

**Fig 4:**
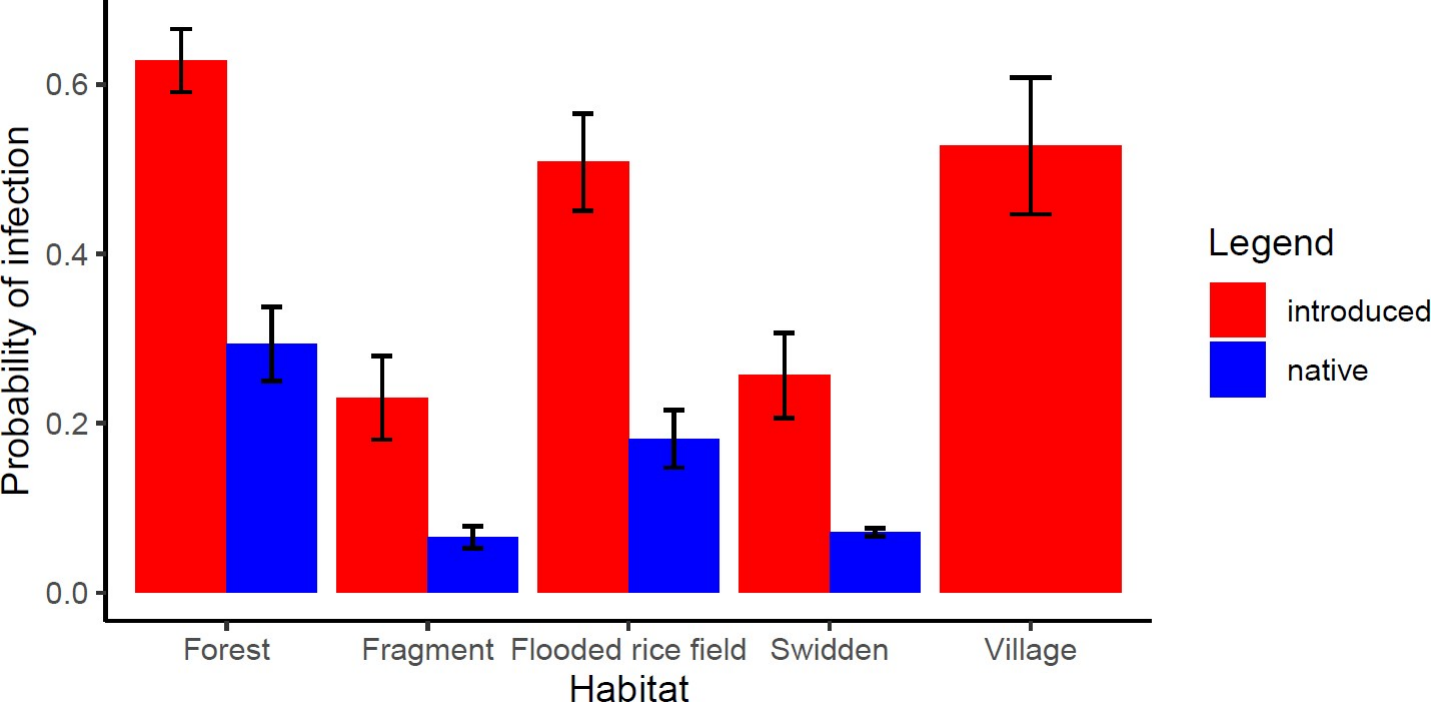
The predicted probability of *Leptospira* infection across habitats and whether the host was native or introduced. The means and standard deviations of the predicted probability of infection based on our model are shown for native and introduced species.

In analyses that examined individual predictors of infection (S6 Table), the model-averaged results show that native species and those individuals captured in forest fragments and the swidden field were less likely to be infected with *Leptospira* than introduced species and individuals in other habitat types (Table 2). The full model explained 18.7% of the variance in *Leptospira* infection. Among habitat types, the animals captured in forest fragments and in the swidden field had significantly lower probability of infection than other habitat types, controlling for all other variables (Fig 4, Table 2). The analyses that incorporated phylogeny found that there was no difference in the probability of infection between native and introduced species and that infection probability was lower in all anthropogenic habitats, controlling for all other variables (lambda value posterior mode = 0.48, 95% highest posterior density = 0.32–0.72, S7 Table).

**Table 2:** Effects of predictor variables on the infection status of individual small mammals. Model-averaged results of model comparisons predicting *Leptospira* infection status. Significant effects are highlighted in italics. These results are based on the models that have a cumulative AIC weight of ≥ 0.95. Note: the following levels were set as the reference (baseline) for comparison: Forest, Granivore, Terrestrial, Female, and Introduced. Coefficients are based on z-score transformation for continuous variables.

Variable	Coefficient (adjusted SE)
Terrestrial/Scansorial	-0.49 (0.34)
*Native*	*-1*.*43 (0*.38)**
Shannon diversity	-0.17 (0.13)
Insectivore	-0.57 (0.45)
Proportion introduced	0.30 (0.30)
Male	0.11 (0.20)
*Fragment*	*-1*.*82 (0*.37)**
Rice field	-0.55 (0.37)
*Swidden*	*-1*.*84 (0*.58)*
Village	-0.86 (0.70)

* p = 0.002

**p<0.001

## Discussion

The prevalence of infection with *Leptospira* was significantly higher for introduced than native species. Introduced species were more likely to be infected than native species, though this result was not significant when controlling for phylogenetic relatedness. The probability of infection with *Leptospira* was lower in forest fragments and swidden fields compared to continuous forest, flooded rice fields, and in the village. Our results illustrate how introduced species and habitat degradation can affect the disease dynamics in this system.

One way that habitat degradation affects biodiversity–and thus disease ecology–is by changing the composition of the host community. In certain cases, parasite transmission increases with habitat degradation when the abundance of competent hosts increases [2,3]. In the classic example of Lyme disease, habitat fragmentation decreased the diversity of mammals and increased the abundance of the highly competent rodent host *Peromyscus leucopus*, which favored the transmission of the associated bacterium [3]. This system and many others led to the hypothesis of the dilution effect, in which habitat degradation reduced diversity and favored competent hosts which increased disease transmission, while diverse habitats ‘diluted’ the competent host populations [4,5,9]. In our study, species diversity was *higher* in some of the disturbed habitats than in natural forest due to the combination of native and introduced species. The negative effects of introduced species on community composition and conservation are diverse [7,13,57], and the effects on disease ecology can be especially significant [7,15]. Introduced species can greatly affect the pool of susceptible hosts when parasites are generalists that can infect multiple host species. It should be noted that native small mammal diversity is notably high in montane habitats on the Marojejy massif [58,59] and comparatively reduced in lowland forests where our sampling was done. Further, because of the rarity of native species, sample sizes for those species were low and future research with larger samples of native species will be needed to determine the robustness of our results. The combination of environmental conditions and host community composition affects the potential for parasite transmission.

When parasites are host-specialists, they are less likely to spill over to a new host, but for generalist parasites, a diverse community is a larger pool of potential hosts [60]. In this study, we expected to find host specificity of leptospires, based the strong host-specificity observed for different *Leptospira* species shed by bats [61] and terrestrial mammals [37,44] endemic to Madagascar. In contrast to our expectations, the results presented here support cross-species transmission for cosmopolitan *Leptospira* lineages. All rats were infected by *L*. *interrogans*, represented by a single *secY* haplotype. This haplotype was found in one specimen of *Setifer setosus* as well as in one specimen of *Mus musculus*. The sequences were identical to that of a *secY* haplotype originally from Kuwait and in *Rattus tanezumi* from Thailand [24]. This haplotype was also identical to that found in *Rattus norvegicus* in eastern Madagascar [35], while it was distinct from that found in the rats sampled on Réunion and Seychelles, which were all infected with the *secY1* haplotype [62,63]. In contrast to *Rattus*, the introduced mammals *Suncus etruscus*, *S*. *murinus*, and all but one *M*. *musculus* were found to share the same *secY*^*3*^ *22* haplotype corresponding to *L*. *kirschneri*. Although *Rattus* were exclusively infected with *L*. *interrogans*, as previously reported in mostly urban settings [35], and *Suncus* and *Mus* almost exclusively infected with *L*. *kirschneri*, our results provide evidence of possible transmission between *Suncus* and *Mus*, and between *Rattus*, *Mus*, and *Setifer*. In contrast to the results presented here, however, native mammals in intact and degraded forests on the island have rarely been found infected with *L*. *interrogans* [23]. Instead, endemic Tenrecidae were found infected by *L*. *mayottensis*, which is endemic to Madagascar and presumably established on Mayotte with the introduction of the tenrecine *Tenrec ecaudatus* to that island [37,42].

The effects of biodiversity and habitat degradation on disease ecology vary among systems. In our study, the probability of infection with *Leptospira* was lowest in the forest fragments and swidden field, but was not significantly different across other habitat types. The animals in the fragments and swidden field may have had lower infection probability because of the lack of water sources within the fragments and field themselves, although streams and seasonally flooded rice fields were within 100 m of the plots. Prevalence and infection probabilities were not higher in flooded rice fields, despite predictions based on the existing knowledge about *Leptospira* transmission. This may be due to variation in the leptospire species found across habitats, since in previous research *L*. *interrogans* appears to be more restricted to humid environments while other species were common across habitats [24]. In Madagascar, lowland valleys are usually cultivated for flooded paddy rice, and introduced rats are abundant in these habitats [64]. The survival and spread of *Leptospira* is facilitated by permanent and seasonally abundant water [65], and environmental contamination is likely enhanced by the mammal hosts that use these habitats. We may not have detected the association between flooded habitats and *Leptospira* infection because of the variation in flooding schedules during this study. In future research, a larger number of plots for each habitat type sampled in both wet and dry seasons will allow us to determine the validity of these results and the drivers of *Leptospira* infection.

Contrary to previous research, where *Leptospira* infection in small mammals captured inside homes was rare [24], we found high prevalence in small mammals in homes. The high incidence of peridomestic species in homes, especially *Mus musculus*, likely enhances the accumulation of leptospires. Because many homes have dirt floors, this permits the leptospires to persist in the soil and high contact rates among abundant small mammals likely increases transmission.

We propose that the *Leptospira* community composition at the Marojejy study site is driven by introduced mammals and their cosmopolitan leptospires (*L*. *interrogans* and *L*. *kirschneri*), which in turn displaced endemic *Leptospira* lineages/species. These transmission dynamics can have important impacts on the native fauna, with unpredictable consequences because the incidence of disease and mortality in wildlife is unknown. However, because we could not obtain *secY* sequences from most endemic mammals, we cannot exclude the existence of endemic lineages/species such as *L*. *mayottensis*, especially in the forest. Future research within the forest, particularly in more montane areas with higher species richness, will help to address whether cosmopolitan *Leptospira* persist far from human disturbed habitats or if there is a transition to endemic *Leptospira* species, as previously reported [41]. We also acknowledge that our models only captured a relatively small proportion of variance in prevalence and infection probability. Future research should investigate other variables that likely play a role including seasonality, more specific quantification of water sources and their flows, and soil moisture [66].

The infection prevalence and *Leptospira* host-specificity pattern described here paves the way for investigations of humans and domesticated animals, with one goal to determine the reservoir of highest epidemiological importance. Leptospirosis is an important zoonotic disease globally [21] and has been reported in Madagascar [38] and neighboring Indian Ocean islands [67]. Malagasy agriculturalists spend long hours working in flooded rice fields, and they commonly use water from fields and neighboring streams and rivers for bathing, cooking and drinking. *Leptospira* can easily be acquired by people when ingesting contaminated water, or via open wounds that are exposed to the water [20]. Thus, people are likely highly exposed to this pathogenic bacterium. One factor that requires future investigation is whether *Leptospira* is detectable in water sources used by people, as well as soils, to determine environmental risk factors for infection [66].

A serological study for the people in this rural village should also be conducted using a Microscopic Agglutination Test based on a panel of isolates including shrew-, mice- and rat-borne *Leptospira* from the field site. Such a public-health study would identify the transmission cycles and address the medical importance of this zoonosis. Determining the intensity of exposure is critical, as a high seroprevalence in humans would increase the awareness of the community to the disease and stimulate early antibiotic (doxycycline) treatment that may significantly reduce disease burden. Results presented here suggest that isolating *Leptospira* from rats and mice is indicative of which leptospires the local human population is exposed. In association with the serological screening of the local population, these data would determine whether the available monovalent or multivalent vaccines are likely to limit infection in local people and domesticated animals [68].

In conclusion, the results of this study highlight the importance of ecological community composition in driving parasite dynamics. Given the high prevalence of *Leptospira* in the system, it is potentially a threat to native biodiversity and to public health. More research is needed to understand the risks for local people of this and other zoonotic transmission pathways. Then, steps can be taken to plan actions that reduce exposure and disease in people.

## Supporting information

S1 Fig Comparison of capture rates across species and habitats. Numbers in parentheses give the number of individuals that tested positive and negative for *Leptospira*. Each point represents the abundance of the species per plot and season. The habitat type of the sites is color coded.
(TIF)

S1 Table Number of individuals captured for which biological samples were obtained by species and season, with totals given. (TXT)

S2 Table Summary of total capture effort (traps + pitfalls). Data are shown for different habitat types across seasons, the total number of exotic and native animals captured, and their capture rates (number captured / number of traps * 1000). This summary includes animals that were released and for which no biological samples were collected, but only used to calculate abundance.
(TXT)

S3 Table Survey effort and capture rate by site, trapping season/year, and species. Column is as follows: W = wet season, D = Dry season, numbers = year of sampling.
(TXT)

S4 Table Raw data on the number of positive and negative samples. Data are given for each species by habitat, site and season, with prevalence, species richness, rarified species richness, Shannon diversity index, whether the species is native or introduced, and their abundance (capture rates).
(TXT)

S5 Table Results of prevalence analysis using the phylogenetically-informed analysis. Analyses were implemented in MCMCglmm. The lambda value, or ‘phylogenetic signal,’ had a posterior mode of 0.47 (95% highest posterior density = 0.28–0.71). Posterior mean effect refers to the mean coefficient estimate from the posterior distribution of samples from the MCMC algorithm, with 5X10^6 generations, omitting the first 1000 as burn-in and sampling every 500 generations. The low and high 95% CI values are confidence intervals from the posterior distribution. ESS = effective sample size, values >200 demonstrate ample sampling of the posterior distribution. p is the probability of the variable having no effect on the dependent variable based on the posterior sample from the MCMC.
(TXT)

S6 Table Data on individual small mammals. Data include their field ID, the season, site, habitat type, species, geographic coordinates, sex (F = female, M = Male), the positive or negative status for Leptospira based on RT-PCR, whether the species was native (N) or introduced (I), and site-level data on the species richness, rarified species richness, and Shannon diversity index. For geographic coordinates at the village site, the animals were captured within individual homes, and the coordinates are low resolution and generalized for the village to protect the identity of participants, according to human ethics protocols.
(TXT)

S7 Table Results of phylogenetically-informed logistic regression of infection. The lambda value, or ‘phylogenetic signal,’ had a posterior mode of 0.48 (95% highest posterior density = 0.32–0.72). The column labels are defined in S5 Table.
(TXT)
