# Supplementary figures and images for: Effects of land use, habitat characteristics, and small mammal community composition on Leptospira prevalence in northeast Madagascar
Source: PLoS Negl Trop Dis. 2020 Dec 31;14(12):e0008946. doi: 10.1371/journal.pntd.0008946 (PMC7774828; doi:10.1371/journal.pntd.0008946)

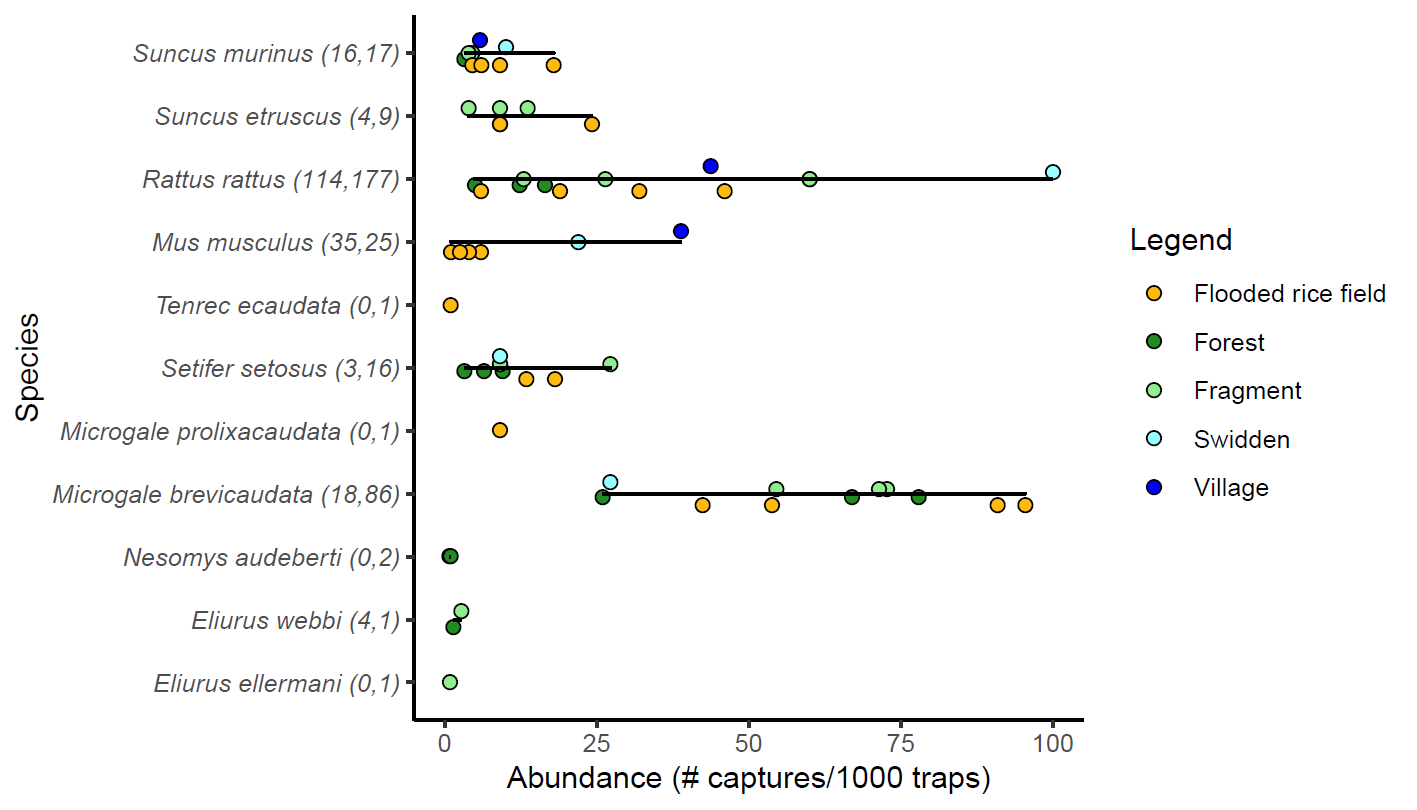

Supplement: S1 Fig — Numbers in parentheses give the number of individuals that tested positive and negative for Leptospira. Each point represents the abundance of the species per plot and season. The habitat type of the sites is color coded. (TIF) [file pntd.0008946.s001.tif]
